# Screening of a Superior *Ganoderma leucocontextum* Strain and Evaluation of Its Agronomic Traits and Bioactive Compounds

**DOI:** 10.3390/foods15213819

**Published:** 2026-10-26

**Authors:** Anyan Fan, Qianying Ren, Baisheng Xu, Aixian Wang, Xiaoyi Ma, Huijuan Sun, Junsheng Fu

**Affiliations:** 1 College of Life Sciences, Fujian Agriculture and Forestry University, Fuzhou 350002, China; 14787054232@163.com (A.F.); 18275663284@163.com (Q.R.); 15092862660@163.com (B.X.); maxiaoyi614@163.com (X.M.); 2 Nanping Institute of Agricultural Sciences, Nanping 354200, China; wangaixian98@163.com; 3 Innovation Center for Edible Fungi Resources and Application Technology, Xizang Autonomous Region, Lhasa 850000, China

**Keywords:** *Ganoderma*, strain identification, ISSR, active ingredients, cultivation adaptability

## Abstract

*Ganoderma leucocontextum*, a new germplasm resource first reported in 2015, has considerable potential for the development of bioactive compounds. However, its wild populations are mainly distributed in high-altitude regions, and its adaptability to cultivation under subtropical conditions in southern China remains insufficiently studied. In this study, seven *G. leucocontextum* strains collected from the Tibet Autonomous Region and Yunnan Province, China, were used as experimental materials. Strain identification and genetic diversity were evaluated using ITS sequence analysis, antagonism assays, and ISSR molecular markers, followed by assessment of resistance to *Trichoderma.* and cultivation performance to screen for a superior strain. The results showed that strain GL2 exhibited superior overall production performance. Under greenhouse cultivation conditions, its fresh fruiting body yield reached 36.07 g per bag, representing a 26.13% increase compared with strain GL1. Under industrial-scale cultivation conditions, the pileus width, the flesh thickness, and fresh weight per bag reached 110.87 mm, 12.06 mm, and 80.46 g, respectively. The triterpene and sterol content of GL2 was 1.04 g/100 g, significantly higher than those of *Ganoderma lucidum* (0.60 g/100 g) and *Ganoderma sinense* (0.68 g/100 g). In addition, its total phenolic content reached 0.37 g/100 g, while total amino acid content was 9.29 g/100 g, both showing clear advantages over the comparison species. All tested strains exhibited moderate resistance to *Trichoderma*, with inhibition rates ranging from 51.28% to 68.79%. Overall, strain GL2 showed stable yield performance, commercial traits, and bioactive component profiles under both protected and industrial cultivation conditions in southern China. It may therefore serve as a promising candidate strain for the cultivation of *G. leucocontextum* in this region and provides a basis for its further development as a functional food ingredient and as a potential alternative resource to traditional *Ganoderma* species.

## 1. Introduction

*Ganoderma lingzhi* is a traditional and highly valued medicinal fungus, known as “immortal grass” in China. It has been used in traditional Chinese medicine for more than 2000 years [1]. The genus *Ganoderma* exhibits rich species diversity, with over 428 known species recorded worldwide [2], and approximately 40 species documented in China [3]. Currently, the major cultivated species include *Ganoderma lucidum*, *Ganoderma sinense*, and *Ganoderma tsugae* [4]. *Ganoderma leucocontextum,* first reported in 2015, represents a newly recognized germplasm resource [5]. One of its most distinctive morphological characteristics is its white to pale-yellow context.

Among various *Ganoderma* species, *G. lucidum* (currently recognized as *G. lingzhi* in modern taxonomy) and *G. sinense* are the most extensively studied and commercially dominant species, and are officially listed in the Pharmacopoeia of the People’s Republic of China [6]. Their derived products are widely used in health foods, pharmaceutical products, and functional food ingredients [7,8,9]. However, different *Ganoderma* species have significant differences in nutritional components and active compounds, and their biological activities also show obvious differences [10]. Mohammadifar et al. [11] found that the total phenols, flavonoids, and betulinic acid content and antioxidant activity of *Ganoderma applanatum* are higher than those of other *Ganoderma* species, while *G. leucocontextum* has a significant advantage in total polysaccharides and total protein content, indicating that the exploration and utilization of species diversity within the *Ganoderma* genus still has broad space. In recent years, increasing evidence has shown that *G. leucocontextum* has unique advantages in bioactive components. Peng et al. [12] systematically compared and found that the protein, ash, flavonoids, and triterpenoid content of *G. leucocontextum* are higher than those of *G. lucidum*, while *G. lucidum* has more dietary fiber and polysaccharides; both have strong antioxidant activity, but *G. leucocontextum* can exert a stronger anti-tumor effect by inducing cell apoptosis, suggesting that it has higher potential for medicinal development. Gao et al. [13] found that the polysaccharide GLP-1 of *G. leucocontextum* activated the immune response of macrophages through the TLR2/Dectin-1 pathway in vivo, could improve immune suppression, and has the prospect of functional food application. Dong et al. [14] used molecular network analysis to isolate and identify 18 new triterpenoid metabolites (Ganoleucocontins A–R) from *G. leucocontextum*, including a class of novel triterpene dimers linked by an HMG moiety (previously unreported). some new compounds showed significant cytotoxicity (*IC*_50_ = 24.39–47.29 μM) to the hepatoma cell line Huh7, and could exert anti-tumor activity by inducing apoptosis and cell cycle arrest. Sun et al. [15] further showed that triterpenoids were major differential metabolites between *G. leucocontextum* and *G. lucidum*, with ganoleucoin-type triterpenoids remaining consistently more abundant in *G. leucocontextum* throughout development, providing new insight into the regulation of triterpenoid biosynthesis. Additional studies have also confirmed the compositional and functional advantages of *G. leucocontextum*. Wang et al. [16] reported that the triterpenoid and polysaccharide contents of *G. leucocontextum* were approximately 20% and 28% higher, respectively, than those of G. lucidum, and its polysaccharides exhibited stronger antioxidant activity. Furthermore, a separate study by Liu et al. [17] demonstrated that Optimization of the cultivation substrate further increased the polysaccharide content of *G. leucocontextum* to 3.19%. In addition, *G. leucocontextum* also has the functions of protecting the kidneys, repairing lung injuries, and improving the pathological conditions of Alzheimer’s disease [18,19,20,21]. Our previous work also achieved progress in factory-based cultivation of *G. leucocontextum*. On-site yield measurements showed that this cultivation mode increased yield by 30.2% compared with traditional greenhouse cultivation, representing a major advance in cultivation technology and providing a practical basis for industrial-scale promotion [22]. Collectively, these findings indicate that *G. leucocontextum* has substantial potential for development as a functional food resource and for industrial cultivation.

Despite these advantages, the cultivation adaptability of *G. leucocontextum* remains an important limitation. Tang et al. [23] found that the “Kangding Lingzhi” strain, originating from Gongga Mountain, Sichuan Province, China, has an optimal growth temperature of 22–25 °C. Yao et al. [24] reported that the new variety “Yunbai Lingzhi”, originating from different regions of Yunnan Province, China, has an optimal growth temperature of 18–23 °C for mycelial growth. These temperature requirements are characteristic of temperate or cool-adapted fungi, which differ markedly from the hot and humid climatic conditions of subtropical regions in southern China. Furthermore, as climate change continues to alter the suitability of agricultural production [25], this further exacerbates the difficulty of geographical expansion and industrial cultivation of *G. leucocontextum* in southern production areas.

To address this limitation, seven *G. leucocontextum* strains were evaluated in the present study. Antagonism tests and ITS sequence analysis were first used to distinguish strains with identical or similar names but different taxonomic identities; Inter-Simple Sequence Repeat (ISSR) molecular markers were employed to assess genetic diversity. On this basis, further *Trichoderma* resistance tests and greenhouse cultivation tests were carried out to select the candidate strain with the best comprehensive performance. The selected strain was then subjected to industrial-scale cultivation, and the contents of polysaccharides, triterpenoids, sterols, polyphenols, flavonoids, and amino acids in fresh fruiting bodies were determined. These analyses were used to evaluate its potential as a functional food ingredient and as an alternative or complementary resource to traditional *Ganoderma* materials. This study aims to provide a scientific basis and germplasm resource support for the cultivation expansion and functional food development of *G. leucocontextum* in southern China.

## 2. Materials and Methods

### 2.1. Tested Strains

Seven strains of *Ganoderma leucocontextum* from different geographic locations were collected as test strains (Table 1). Fruiting experiments of *G. leucocontextum* were conducted under protected cultivation in Fujian, China.

### 2.2. Culture Medium

Based on the method described by Cai et al. [26] with slight modifications, the following media were prepared. The PDA-enriched medium contained 200 g of peeled potatoes, 20 g of agar (Fujian Jinyan Marine Biological Technology Co., Ltd., Quanzhou, China), 20 g of glucose, 2 g of KH_2_PO_4_, 1.5 g of MgSO_4_, 10 mg of vitamin B_1_ and 5 g of tryptone (the latter five reagents from Sinopharm Chemical Reagent Co., Ltd., Shanghai, China), with distilled water added to a final volume of 1 L and the pH adjusted naturally. The liquid medium had the same composition but without agar. The cultivation substrate consisted of 78% wood shavings, 20% wheat bran, 1% glucose, and 1% gypsum, with pH adjusted naturally; the wood shavings, wheat bran, and gypsum were provided by Shaxian Jinnong Mushroom Industry Co., Ltd. (Sanming, China).

### 2.3. Isolation of Strains from Tissue Samples

Following Stamets’ [27] method for isolating fruiting body tissue, robust, intact, and pest- and disease-free *G. leucocontextum* fruiting bodies were selected. After washing to remove surface dust and impurities, the surface was disinfected with 75% alcohol (Sinopharm Chemical Reagent Co., Ltd., Shanghai, China). The specimens were then transferred to a sterile workbench (VS-1300-U clean bench was supplied by Antai Air Technology Co., Ltd., Suzhou, China), where the outer edge of the cap was excised using a flame-sterilized scalpel. Young tissue blocks (approximately the size of a soybean) were inoculated at the center of the PDA medium. This procedure was repeated, and the samples were incubated at 25 °C in an incubator (Shanghai Boxun Industry & Commerce Co., Ltd. Medical Equipment Factory, Shanghai, China) to observe mycelial growth.

### 2.4. DNA Extraction

Under aseptic conditions, a hole puncher (Nantong Kewashi Laboratory Equipment Co., Ltd., Nantong, China) was used to make holes along the edge of the colony of the each test strain (the area where the mycelium grows vigorously) to collect mycelial blocks. These mycelial plugs were then inoculated onto PDA medium covered with sterile cellophane membranes. When the mycelium reached two-thirds of the surface of the glass paper, it was scraped and transferred to a sterilized mortar. Liquid nitrogen (Xiamen Honghua Industrial Gas Co., Ltd., Xiamen, China) was added to thoroughly grind it into powder, and the powder was aliquoted into 1.5 mL centrifuge tubes. The UNIQ-10 column-type fungal genomic DNA extraction kit obtained from Sangon Biotech (Shanghai) Co., Ltd., Shanghai, China; (LOT: JB27KA2004) was used to extract the genomic DNA of each test strain according to the manufacturer’s instructions.

### 2.5. Identification of ITS Genes

The ITS region of the genomic DNA of the test strains was amplified by PCR using the universal primers ITS1 (5′-TCCGTAGGTGAACCTGCGG-3′) and ITS4 (5′-TCCTCCGCTTATTGATATGC-3′) [28], the primers were synthesized by Sangon Biotech (Shanghai) Co., Ltd. The PCR reaction mixture (total volume 25 μL) contained: 12.5 μL of 2× Master Mix (Fuzhou Nanjiang Biotechnology Co., Ltd., Fuzhou, China), 1 μL of each primer ITS1 and ITS4, 1 μL of template DNA, and 9.5 μL of ddH_2_O. PCR amplification was performed on a VQ 96 Fast gradient thermal cycler (Monad Biotech Co., Ltd., Suzhou, China). The amplification procedure was as follows: 5 min of pre-denaturation at 95 °C; 30 s of denaturation at 95 °C, 30 s of annealing at 58 °C, and 90 s of extension at 72 °C, for a total of 35 cycles; followed by a final extension at 72 °C for 7 min. The amplified products were detected by 1% agarose gel electrophoresis, and 2 μL of the PCR product was loaded for analysis. If a single bright band appeared, it indicated successful amplification. The remaining PCR products were entrusted to Shenggong Biotechnology Engineering (Shanghai, China) Co., Ltd. (Fuzhou, China) for sequencing. The ITS-rDNA sequences were compared using the Basic Local Alignment Search Tool (BLAST) on the NCBI website (URL: http://www.ncbi.nlm.nih.gov/, accessed on 12 July 2026). The most homologous reference sequence was downloaded, and a phylogenetic tree was constructed using the Maximum Likelihood (ML) method in MEGA 11.0 software.

The primers ITS1 and ITS4 were synthesized by Sangon Biotech (Shanghai) Co., Ltd. Master Mix (Shanghai, China). PCR amplification was performed on a MyCycler thermal cycler (Bio-Rad Laboratories, Hercules, CA, USA). Electrophoresis was carried out using a DYY-8C electrophoresis apparatus (Beijing Liuyi Instrument Factory, Beijing, China).

### 2.6. Antagonism Test

Under aseptic conditions, a hole-making tool is used to make holes along the edge of the test strain colonies to collect mycelial blocks. These mycelial blocks are then inoculated in pairs in the center of the PDA plates (with a spacing of 2–5 cm) and placed in an incubator at 25 °C in the dark for cultivation. Each antagonistic combination is set up with 3 biological replicates. The junctions of the colonies are observed regularly. If a furrow-shaped, raised-shaped, or isolated-type antagonistic line appears, it indicates that there are significant genetic differences between the two strains.

### 2.7. Genetic Diversity Analysis

#### 2.7.1. Primer Selection

According to Xu et al. [29] and Zhao et al. [30], 20 ISSR primers were selected and synthesized by Sangon Biotech (Shanghai, China) Co., Ltd. (Fuzhou, China). Detailed primer information is provided in Table 2.

#### 2.7.2. ISSR Amplification

Use an amplification system of 20 μL: 12.5 μL of 2× Master Mix, 1 μL of ISSR primers, 1 μL of template DNA, and 10.5 μL of ddH_2_O.

Amplification reaction procedure: 95 °C for 5 min, 95 °C for 30 min, 48 °C for 30 s, 72 °C for 1 min, 35 cycles, 72 °C for 10 min, then stored at 4 °C.

#### 2.7.3. Agarose Gel Electrophoresis

Using a 2% agarose gel, electrophoresis was performed at a voltage of 100 U for 40 min and then observed and photographed on an ultraviolet gel imaging system.

#### 2.7.4. Data Analysis

The amplification electrophoresis data were scored manually, with clear bands at the same migration position recorded as ‘1’ and the absence of bands as ‘0’, to establish a binary (0–1) matrix [29]. The UPGMA dendrogram was then constructed using the statistical software NTSYS-pc 2.10e.

### 2.8. Evaluation of Trichoderma Resistance

Based on the methods of Cao et al. [31] and Umar et al. [32], with some modifications, in a sterile laminar flow hood, use a 5 mm diameter puncher to take a piece of mycelium along the edge of the test strain colony, and inoculate it at a position 1.5 cm away from the center of the PDA plate. Cultivate for 4 d. Subsequently, inoculate the vigorous *Trichoderma* mycelium piece (5 mm in diameter) at the symmetrical position 2.5 cm away from the center. Place the plate in a 25 °C constant-temperature incubator for inverted cultivation. When the mycelium of the two colonies comes into contact, observe the antagonistic line morphology at the junction; at the same time, using the cross method, measure the growth length of the *G. leucocontextum* mycelium from the edge of the *Trichoderma* block to the junction line, and record it as *A*_1_ (mm). The control group (CK) does not inoculate *Trichoderma*, and the growth length of the *G. leucocontextum* mycelium from the edge of the inoculation block to the junction line is measured at the same incubation time and recorded as *A*_0_ (mm). See Equation (1) for the calculation formula. Each treatment is set up with 3 replicates.

Inhibition rate (%) = (*A*_0_ − *A*_1_)/*A*_0_ × 100% (1)

### 2.9. Facility-Based Mushroom Cultivation Experiment

#### 2.9.1. Preparation of Cultivated Strains

The PDB medium was divided into 250 mL beakers, with each beaker containing 100 mL. After being sterilized in an LDZF-50L vertical pressure steam sterilizer (Shanghai Shen’an Medical Instrument Co., Ltd., Shanghai, China) at 121 °C for 30 min and then cooled, the medium was ready for use. In the ultra-clean workbench environment, a 5 mm diameter puncher was used to make holes at equal intervals on the colonies of the test strains. Then, complete bacterial patches were picked and transferred to the PDB medium. Five mycelial plugs were placed in each beaker, and the bottle mouths were sealed with sealing films. Finally, the medium was incubated at 25 °C with shaking at 160 r/min in the dark for 7 d using a BSD-YX2600 vertical intelligent precision shaker (Shanghai Boxun Medical Biological Instrument Corp., Shanghai, China).

#### 2.9.2. Preparation of Mushroom Bags and Cultivation of Mushrooms

The production of the mycelial packs follows the cultivation substrate formula described in Section 2.2. The cultivation substrate is proportionally mixed and evenly distributed into 17 cm diameter edible fungus cultivation bags. The bags were sterilized at 121 °C for 3 h, then cooled and inoculated on a clean bench. The inoculated mycelial packs were moved to the cultivation room for mycelium growth management and mushroom production management. The growth rate of the mycelial packs, the number of days until the bags are full, the number of days for primordia formation, and the agronomic traits of the mature fruiting bodies were recorded. A facility-based mushroom cultivation experiment was conducted from September 2025 to January 2026 at Shaxian Jinnong Mushroom Industry Co., Ltd. (Sanming, China) under greenhouse conditions. For each strain, 30 bags were prepared and randomly arranged in the greenhouse (totaling 210 bags for the seven strains). The growth rate of the mycelial packs, the number of days until the bags were full, the number of days for primordia formation, and the agronomic traits of the mature fruiting bodies were recorded.

### 2.10. Factory-Based Mushroom Cultivation Verification Experiment

The methods for preparing the cultivation strains and making the fungal bags were the same as those in Section 2.9.1 and Section 2.9.2. Mushroom cultivation experiments were conducted in a factory environment. Based on the preliminary screening in the facility-based cultivation (Section 2.9.2), strain GL2 exhibited superior comprehensive performance and was selected for the factory-based validation. The factory-based cultivation experiment was conducted from March 2026 to July 2026 at the same facility (Shaxian Jinnong Mushroom Industry Co., Ltd., Sanming, China). A total of 2000 bags of GL2 were prepared for a single large-scale cultivation trial. During fungal management, the cultivation temperature was 25–28 °C, and the fungi were cultivated in the dark while contaminated fungal bags were promptly removed and handled. Mushroom cultivation management was carried out under conditions of a temperature of 22–28 °C and an air humidity of 80–90%, and the agronomic traits of the fruiting bodies were recorded.

### 2.11. Determination of Nutritional Components of Fresh Fruiting Bodies

#### 2.11.1. Sample Processing

Collect 300 g of fresh young fruiting bodies of high-quality GL2 and place them in a 50 °C electrically heated constant-temperature blower drying oven to dry until the mass remains constant. Then, store them in an airtight container at 4 °C for future use. *Ganoderma lucidum* (‘Fuzhi 94’) and *Ganoderma sinense* (‘Wuzhi 8’) were used as reference materials.

#### 2.11.2. Amino Acid Content (AAC)

The tests were conducted by Huatai Testing and Certification Group Co., Ltd. (Fuzhou, China), and each indicator was measured three times in duplicate. The AAC test was carried out in accordance with GB5009.124—2016 [33].

### 2.12. Determination of Active Components of Fresh Fruiting Bodies

Sample processing was performed as described in Section 2.11.1. The testing was conducted by Huatai Testing and Certification Group Co., Ltd. (Fuzhou, China), and each indicator was measured three times. Among them, the contents of polysaccharides, triterpenoids, and sterols were determined following the corresponding methods in Volume I of the Pharmacopeia of the People’s Republic of China (2020 Edition); the total polyphenol content was determined in accordance with the group standard T/AHFIA 005-2018 [34]; and the total flavonoid content was determined in accordance with the “Technical Guidance Principles for Physical and Chemical and Hygiene Indicators of Health Food (2020 Edition)”.

#### 2.12.1. Polysaccharide Content (PC)

A 2.0 g portion of the sample powder was extracted using the water extraction and alcohol precipitation method. Water (60 mL) was added, and the mixture was allowed to stand for 1 h, then heated under reflux for 4 h and filtered while hot. The residue was refluxed again with 60 mL of water for 3 h. The filtrates were combined and evaporated to dryness in a water bath. The residue was dissolved in 5 mL of water, and 75 mL of ethanol was slowly added with stirring. The mixture was allowed to precipitate at 4 °C for 12 h. After centrifugation, the supernatant was discarded, and the precipitate was redissolved in hot water. The solution was transferred to a 50 mL volumetric flask and diluted to the mark. The solution was centrifuged again, and 3 mL of the supernatant was transferred to a 25 mL volumetric flask and diluted with water to the mark to obtain the test solution. Finally, the anthrone–sulfuric acid method was used to measure the absorbance at 625 nm, and the PC was calculated using the standard curve [35].

#### 2.12.2. Total Triterpene Content (TTC) and Sterol Content (SC)

The TTC and SC were prepared by an ultrasonic-assisted ethanol extraction method. The sample powder (2 g) was placed in a stoppered conical flask and mixed with 50 mL of ethanol. The mixture was subjected to ultrasonic treatment (power 140 W, frequency 42 kHz) for 45 min and then filtered. The filtrate was transferred to a 100 mL volumetric flask, and the filter residue was washed with an appropriate amount of ethanol. The wash liquid was combined into the same volumetric flask, diluted to the mark with ethanol, and shaken well to obtain the test sample solution. Ultraviolet–visible spectrophotometry was used to prepare a standard curve with oleanolic acid as the reference substance. The absorbance was measured at a wavelength of 546 nm, and the TTC and SC were calculated [35].

#### 2.12.3. Total Phenolic Content (TPC)

The TPC was prepared by an ultrasonic-assisted ethanol extraction method. The extract (5 mL) was placed in a 100 mL flask, mixed with 30 mL of 60% ethanol, and subjected to ultrasonic treatment for 10 min. The solution was then diluted to 50 mL with 60% ethanol, shaken well, and filtered for subsequent analysis. Ultraviolet–visible spectrophotometry was used to prepare a standard curve with gallic acid as the reference substance. Aliquots (0.0, 0.2, 0.4, 0.6, 1.0, and 1.5 mL) of the gallic acid standard stock solution (200 mg/L) were transferred into 10 mL volumetric flasks and diluted to the mark with 60% ethanol. Subsequently, 1.0 mL of each dilution was mixed with 2.5 mL of Folin–Ciocalteu reagent, shaken, and combined with 2.5 mL of 15% Na_2_CO_3_ solution. The mixture was diluted to volume with water, incubated in a 40 °C water bath for 60 min, and cooled for 20 min. The absorbance was measured at a wavelength of 778 nm, and the content of TPC was calculated based on the standard curve [34].

#### 2.12.4. Total Flavonoid Content (TFC)

The TFC was extracted and purified using an ethanol extraction and polyamide adsorption method. Briefly, a certain amount of sample powder was mixed with ethanol in a 25 mL volumetric flask, shaken, and ultrasonicated for 20 min. After standing, 1.0 mL of the supernatant was pipetted into an evaporating dish, mixed with 1 g of polyamide powder for adsorption, and evaporated to dryness in a water bath. The mixture was then transferred to a chromatography column and sequentially eluted with 20 mL of toluene (eluate discarded) and methanol. The methanol eluates were collected and diluted to 25 mL to obtain the test solution. The total flavonoid content was determined by UV–Vis spectrophotometry using rutin as the reference standard. The absorbance was measured at 360 nm, and the content was calculated based on the standard curve [36].

### 2.13. Statistical Analysis

The experimental data were processed using IBM SPSS 26.0 and GraphPad Prism 11.0.1 software. Comparisons between different treatment groups were conducted using *t*-tests, with *p* < 0.05 indicating a significant difference and *p* < 0.01 indicating an extremely significant difference.

## 3. Results

### 3.1. Identification of Strain

**Figure 1:**
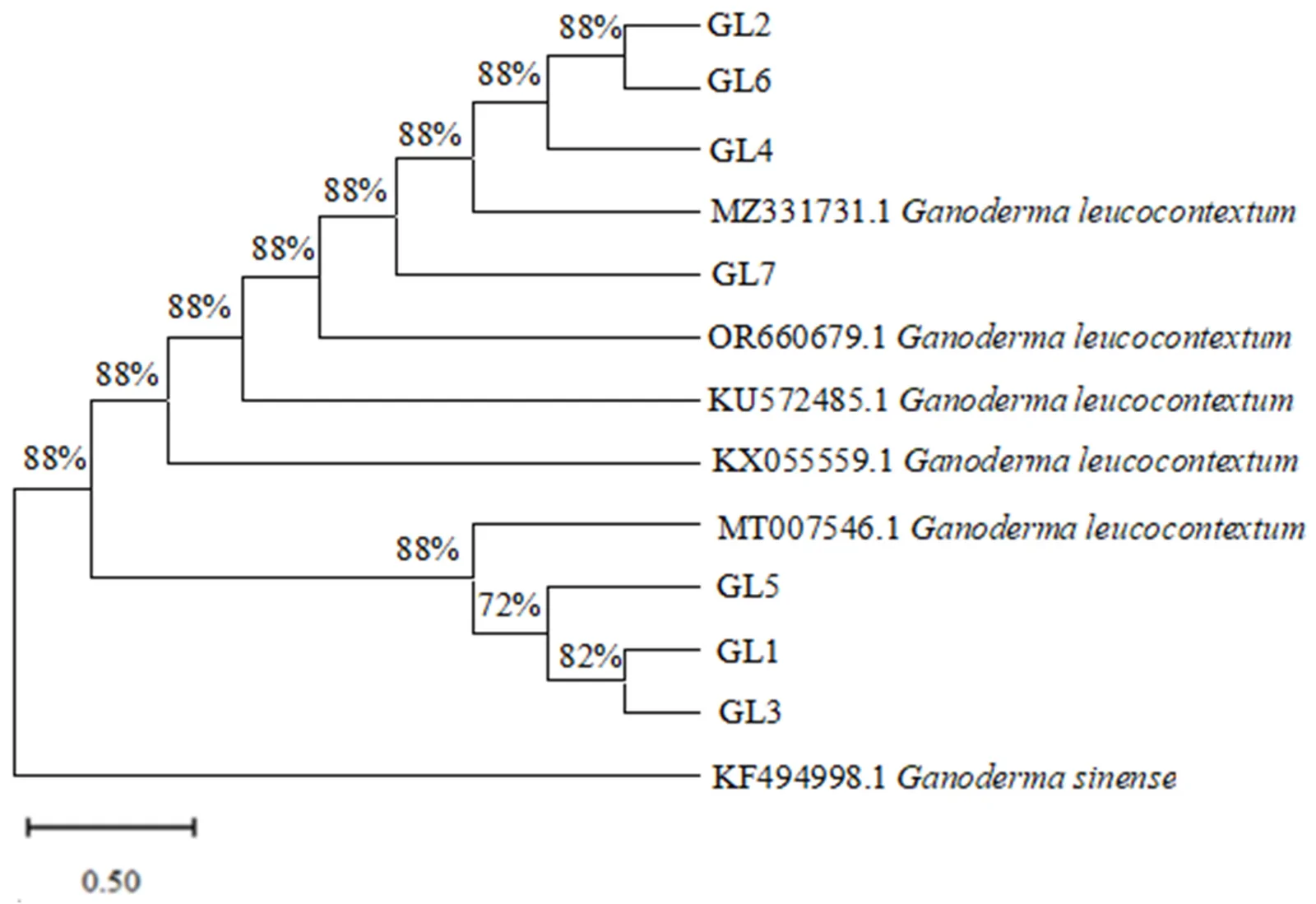
Phylogenetic tree of seven *G. leucocontextum* strains based on ITS sequences.

Amplification of the ITS region using the ITS1/ITS4 primer pair yielded target fragments of approximately 750 bp for all seven test strains. After sequencing and sequence assembly, high-quality ITS sequences were obtained and subjected to BLAST analysis against the NCBI database. A maximum-likelihood phylogenetic tree was subsequently constructed, with *Ganoderma sinense* used as the outgroup (Figure 1). The phylogenetic analysis showed that all seven strains were identified as *Ganoderma leucocontextum*. However, the seven *G. leucocontextum* strains did not cluster into a single clade but were distributed among several secondary branches, indicating a certain degree of intraspecific genetic differentiation. These results confirmed a reliable molecular basis for subsequent genetic diversity analysis.

### 3.2. Results of Antagonism Test

**Figure 2:**
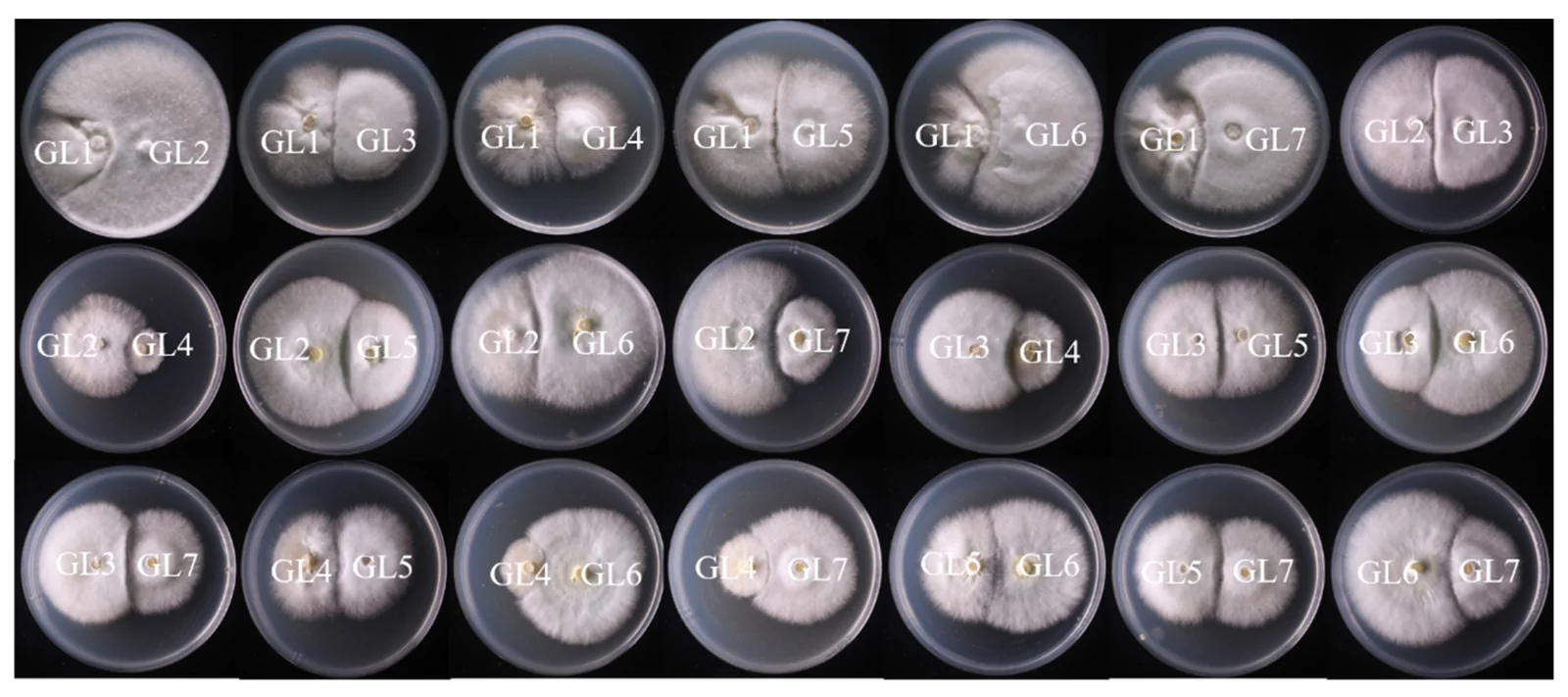
Antagonistic interactions among the seven *G. leucocontextum* strains.

Antagonistic experiments were conducted between the mycelia of seven *G. leucocontextum* strains to identify the germplasm resources. Based on the morphological characteristics observed at the contact zones between opposing colonies, the antagonistic results could be roughly classified into three types: protrusion, furrow, and linear types. As shown in Figure 2, a distinct mycelial protrusion developed at the interaction zone between GL1 and GL2, indicating a pronounced antagonistic reaction. In GL2-GL7 pairing, a clear furrow formed at the colony interface, whereas a continuous and well-defined boundary line was observed between GL1 and GL3. Both patterns also indicated evident antagonistic reactions. Based on these morphological categories, the pairwise antagonistic responses among the seven strains were systematically recorded and summarized in Table 3.

As shown in Table 3, the seven tested strains exhibited distinct patterns of antagonistic reactions. A total of 21 pairwise combinations were evaluated, including 4 combinations showing protrusion-type antagonism, 11 combinations showing furrow-type antagonism, and 6 combinations showing linear-type antagonism. The presence of antagonistic reactions in all pairwise combinations indicated discernible differences among the seven strains and supported their discrimination as distinct germplasm resources. 

### 3.3. Analysis of Genetic Diversity Results

**Figure 3:**
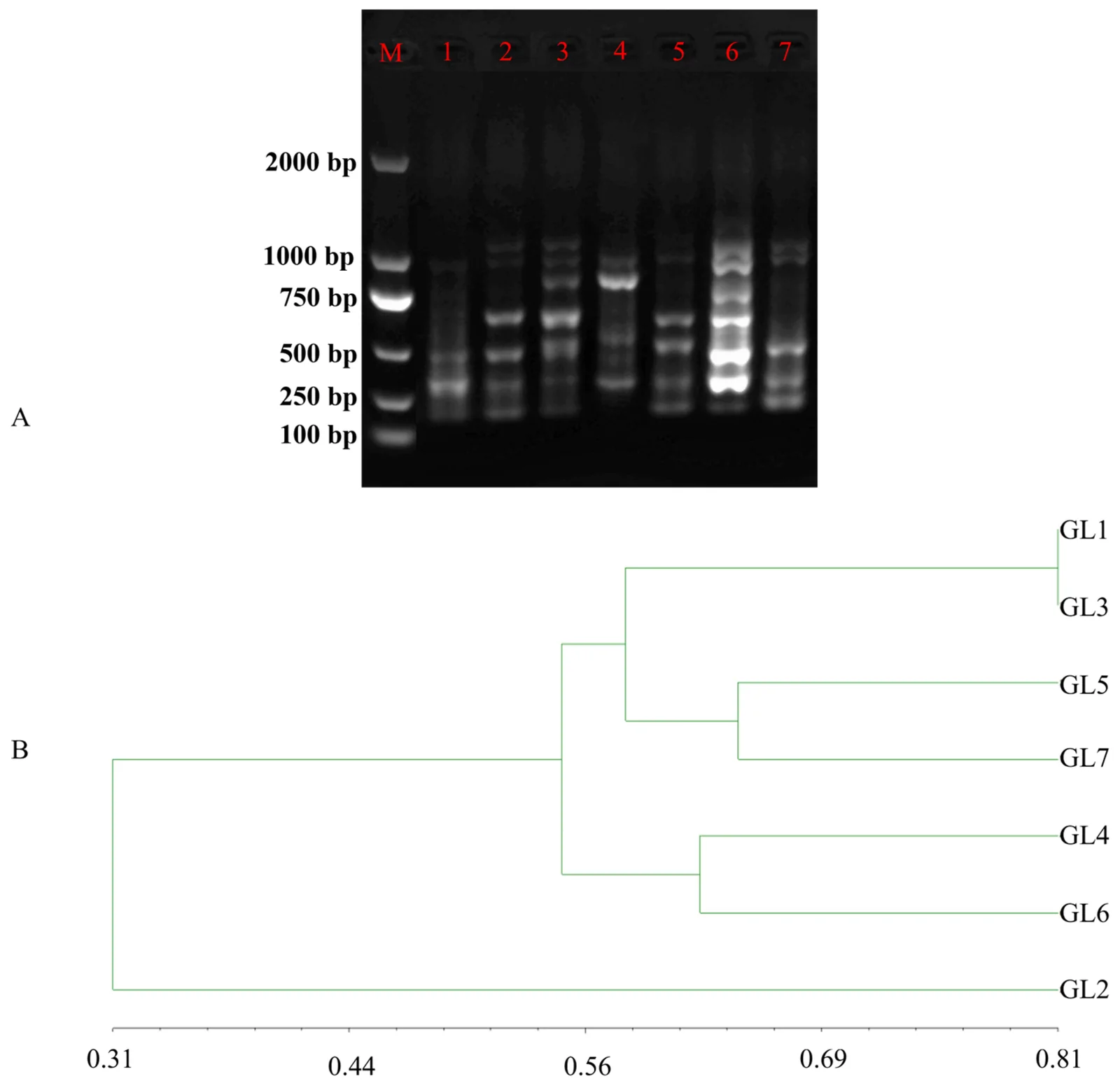
Genetic diversity analysis of seven *G. leucocontextum* strains based on ISSR markers. (**A**) ISSR amplification profiles of seven *G. leucocontextum* strains using primer 15. (**B**) ISSR cluster analysis of the seven *G. leucocontextum* strains.

Among the 20 ISSR primers initially screened, 5 primers (ISSR1, ISSR15, ISSR16, ISSR18, and ISSR19) were selected based on their reproducible amplification, clear banding patterns, and high polymorphism. These 5 primers were used to perform ISSR-PCR amplification on the test strains, and the amplification patterns are shown in Figure 3A. A total of 60 reproducible bands were amplified, with fragment sizes ranging from 200 to 1100 bp, of which 49 were polymorphic. The overall percentage of polymorphic bands was 81.67%, indicating a relatively high level of genetic diversity among the tested strains.

Cluster analysis was conducted on seven strains, and the genetic clustering diagram is shown in Figure 3B. The ISSR analysis indicated that the genetic similarity coefficients of the seven test strains ranged from 0.31 to 0.81. GL2 was relatively far from other strains in terms of genetic distance, with a similarity coefficient of 0.31, and was clustered into a single group; the similarity coefficient between GL1 and GL3 was 0.81, indicating that these two strains had a closer genetic relationship; the similarity coefficient between GL5 and GL7 was 0.64; the similarity coefficient between GL4 and GL6 was 0.61. When the genetic similarity coefficient was 0.58, the test strains could be divided into four genetic clustering groups: strain GL1 and GL3 were clustered into group I; strain GL5 and GL7 were clustered into group II; strain GL4 and GL6 were clustered into group III; and strain GL2 was clustered alone as group IV.

### 3.4. Results of Trichoderma Resistance

As shown in Table 4, dual-culture antagonism assays were performed on agar plates to evaluate the resistance of the seven *G. leucocontextum* strains to *Trichoderma*. The inhibition rates of *Trichoderma* against the tested strains ranged from 51.28% to 68.79%. Among them, *Trichoderma* had the highest inhibition rate against GL6 (68.79%), followed by GL4 (68.42%) and GL5 (66.28%), with the lowest inhibition rate against GL3 (51.28%). The inhibition rates against GL2, GL4, GL5, and GL6 were significantly higher than those against GL3 (*p* < 0.05), while GL7 (59.84%) showed no significant difference compared to the other strains. This result indicated that the antagonistic abilities of these seven strains towards *Trichoderma* were generally comparable, possibly belonging to a medium level.

**Figure 4:**
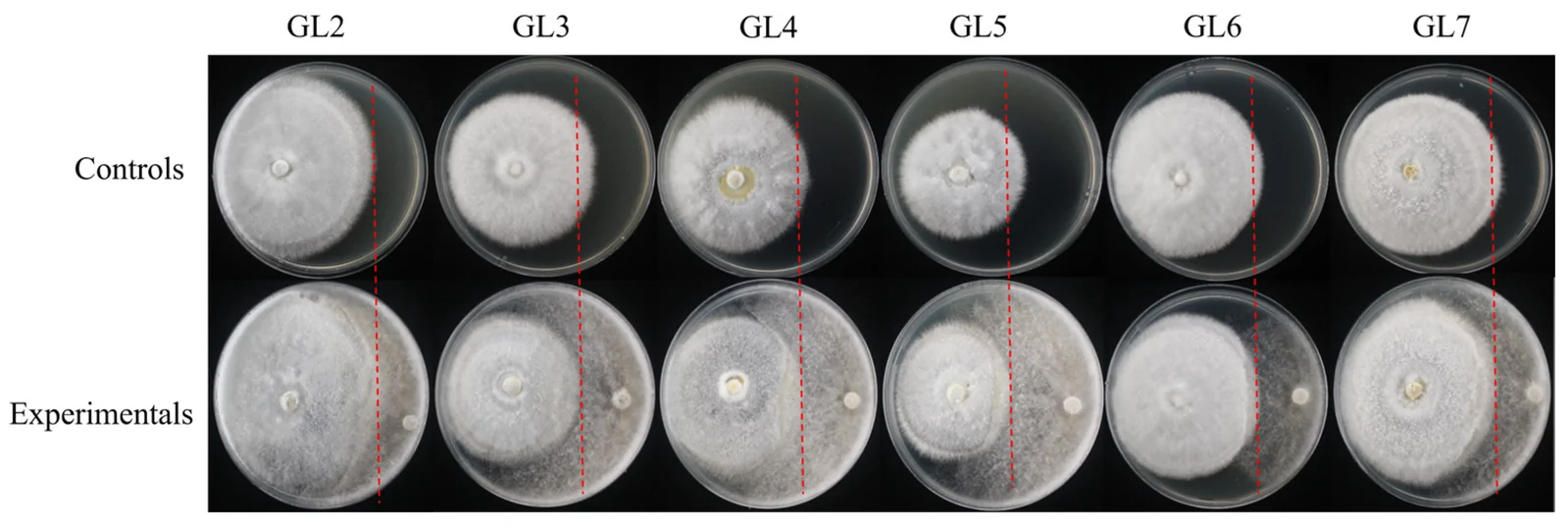
Inhibitory effects of *Trichoderma* on seven *G. leucocontextum* strains. The red dashed lines indicate the colony edge of the mycelial growth after mold inoculation. In the control group, the portion extending beyond the red dashed line represents the normal mycelial growth of the tested strain, which is inhibited by the mold in the experimental group.

The corresponding morphological characteristics of the antagonistic effects are presented in Figure 4. Although the quantitative data showed no significant differences, GL3 and GL7 exhibited a slight advantage at the same level, but further verification of their potential biocontrol value is needed through larger sample repeated experiments or combined with other antagonistic indicators, such as the width of the inhibitory zone and the activity of volatile metabolites. Overall, the inhibition rate variation in Trichoderma against this batch of *G. leucocontextum* strains was not significant. When screening for strong antagonistic strains, it is necessary to expand the source of strains or optimize the cultivation conditions to improve discrimination.

### 3.5. Experimental Results of Facility Mushroom Cultivation

#### 3.5.1. Evaluation Results of Yeast Packet Growth Characteristics

**Figure 5:**
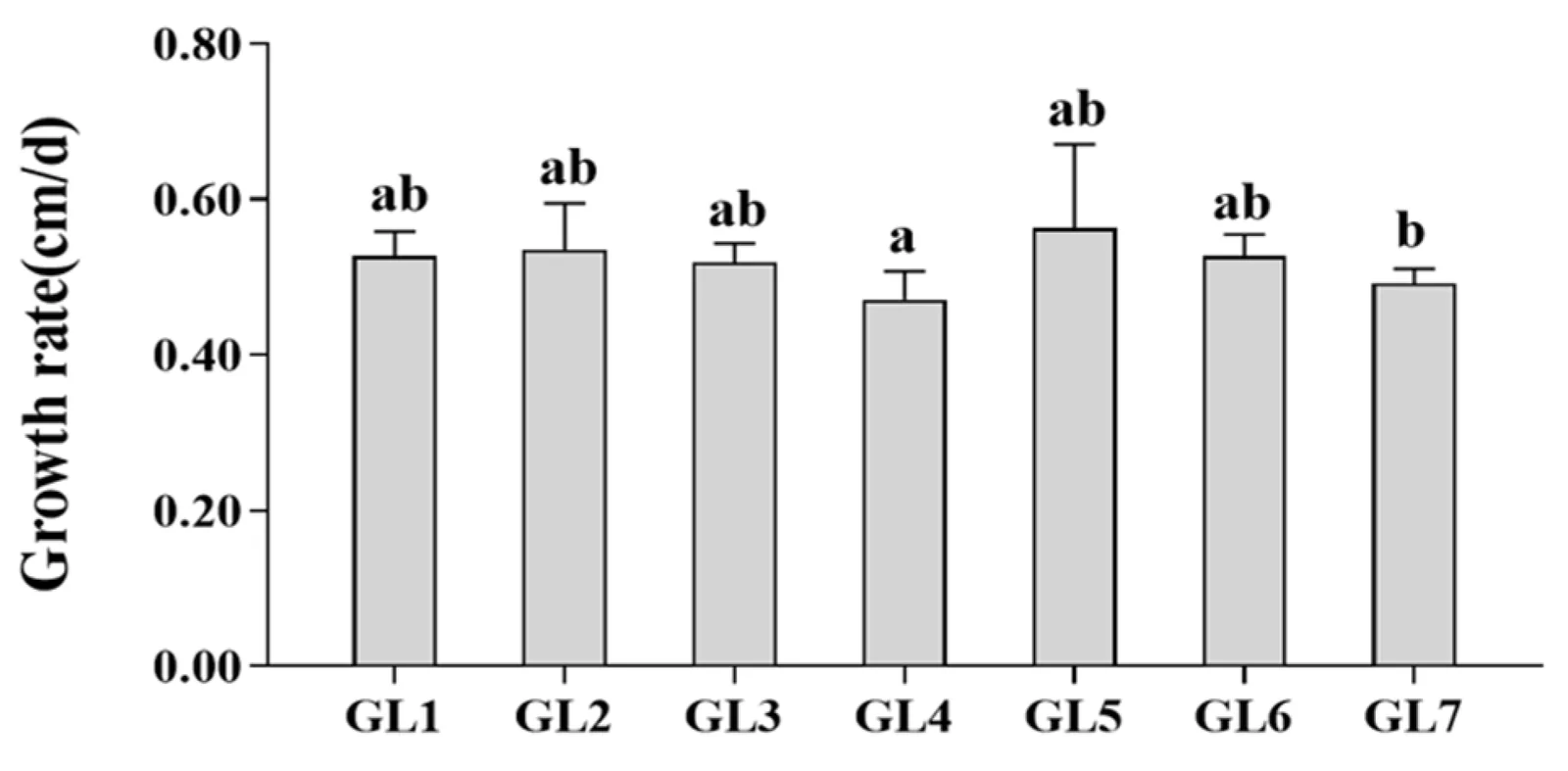
Mycelial growth rates of seven *G. leucocortextum* strains on spawn bags. Means with different letters indicate significant differences (*p* < 0.05).

The growth characteristics of mycelium and the differentiation of primordia of seven strains of *G. leucocontextum* were compared and analyzed. The mycelial growth rates on spawn bags are shown in Figure 5, and the times to full bag colonization and primordium formation are summarized in Table 5. The growth rate of mycelium in the cultivation bags ranged from 0.47 to 0.56 cm/day (Figure 5). GL5 had the highest growth rate (0.56 cm/day), while GL4 had the lowest (0.47 cm/day), and the other strains ranged from 0.49 to 0.53 cm/day. In the cultivation bags, the shortest time for the bags to be filled with mycelium was GL2 (30 d), followed by GL3 (31 d) and GL6 (33 d), while GL1, GL4, GL5, and GL7 took 35 d (Table 5). It is worth noting that the strain with the highest mycelium growth rate, GL5, did not show a shorter filling time, indicating that the mycelium spread speed within the bag was not completely uniform, which may be related to the inoculation method, the compactness of the substrate, and the distribution of oxygen, etc.

In terms of the formation time of primordia, the variation range of each strain was large, ranging from 38 to 45 d. GL1 and GL3 were the earliest to form primordia (38 d), followed by GL2 (39 d) and GL7 (40 d), while GL4, GL5, and GL6 were the latest (45 d). Overall, the interval between filling the bag and the appearance of primordia varied significantly among the different strains: GL1 and GL3 flowered only 3–7 d after filling the bag, while GL4, GL5, and GL6 required 10–12 d, indicating that the latter three strains had a delay in the transition from nutritional growth to reproductive growth. Moreover, although GL2 had the fastest filling time (30 d), its primordia formation was later than GL1 and GL3, suggesting that fast-growing mycelium may not necessarily promote early mushroom production. In conclusion, all seven strains exhibited a clear intraspecific variation in mycelial growth, substrate colonization, and the timing of primordium differentiation.

#### 3.5.2. Comparative Fruiting Results of Different Strains

**Figure 6:**
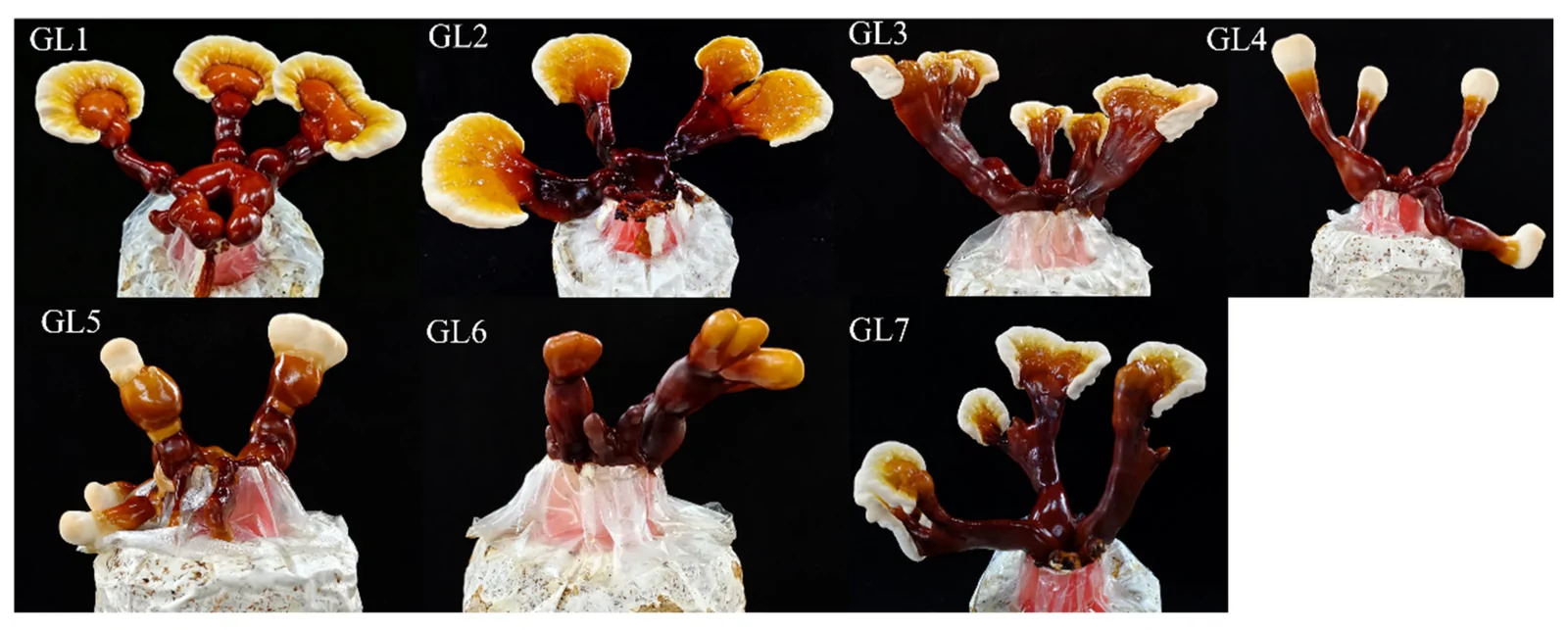
Mycelial growth rates of seven *G. leucocortextum* strains on spawn bags.

As shown in Figure 6, the mushroom formation of the seven tested strains showed significant differentiation, and there were significant differences in the mushroom formation performance of the tested strains. Among them, the two strains GL1 and GL2 could normally form fruiting bodies, and their appearance developed completely; GL3 and GL7 could complete mushroom formation, but the mushroom caps were limited in opening, and the mushroom stems were relatively elongated, resulting in an overall poor commercial form. The three strains GL4, GL5, and GL6 had a delayed development process and only reached the primordium differentiation stage, failing to form mushroom caps. Further assessment of the normal performance of GL1 and GL2 regarding fresh mushroom yield and appearance quality showed that the average fresh weight of single bags of GL1 was 28.60 g, with a mushroom cap that was semi-circular to nearly kidney-shaped, having a distinct lacquer-like luster, with a color between yellowish-brown and reddish-brown, and the mushroom stems were mostly lateral or proximal, with medium length and a slightly thin diameter, and a firm texture.

In contrast, the single-bag fresh weight of GL2 was higher, at 36.07 g; its mushroom cap was large and thick, with a shape close to circular or kidney-shaped, having a bright surface luster, a color of red-brown to dark brown-red, with rounded edges and a light-colored ring pattern; the mushroom stems were thick and mostly lateral or proximal, with an appropriate length and good connection with the mushroom cap, and the overall appearance quality was better than that of GL1. In summary, strain GL2 exhibited the best mushroom formation performance under summer cultivation conditions in Fujian, China, indicating its potential as a candidate strain for the local cultivation of *G. leucocontextum*.

### 3.6. Experimental Results of Factory-Farmed Mushroom Cultivation

**Figure 7:**
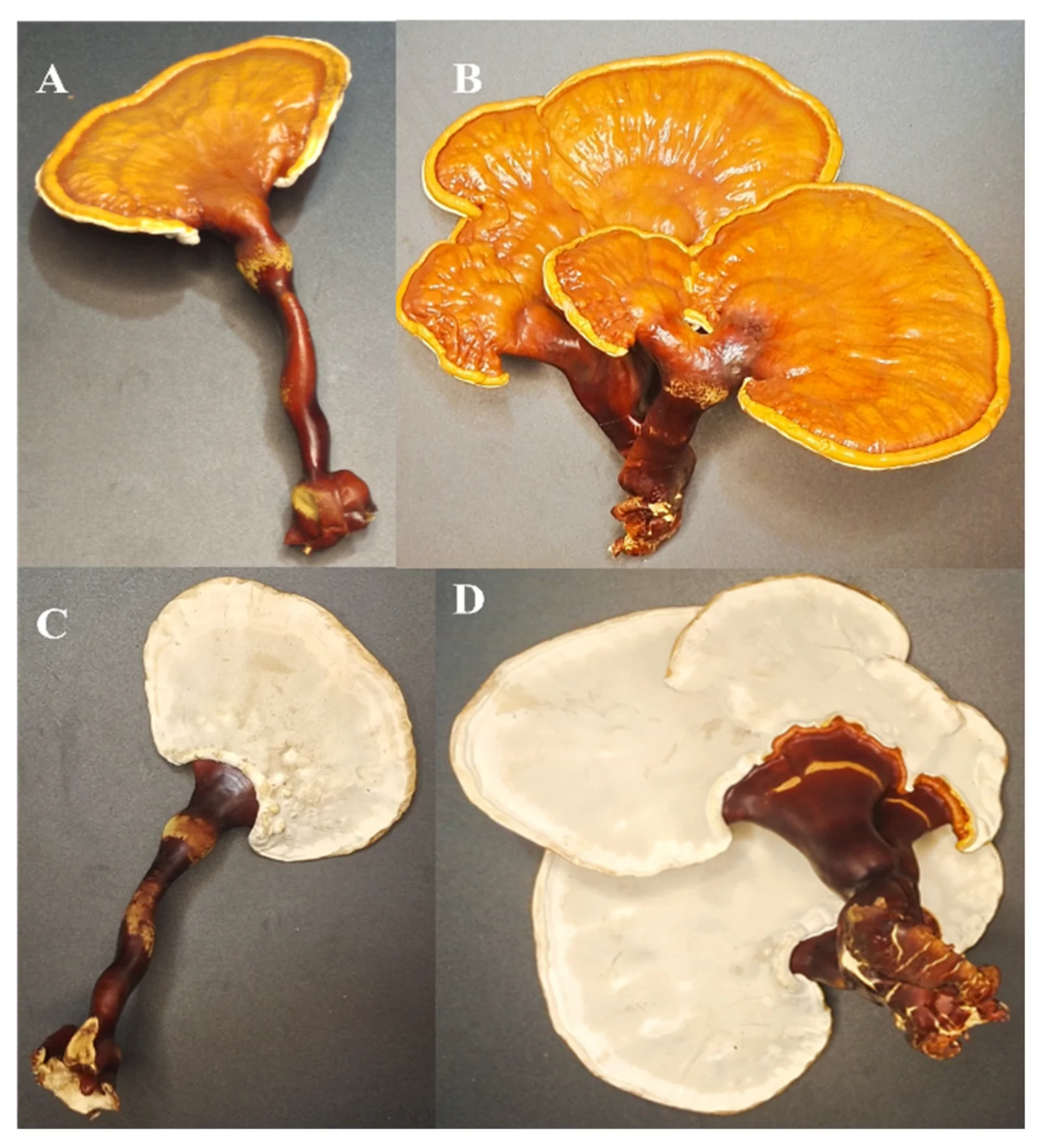
Fruiting bodies of GL2 under industrial cultivation. (**A**,**C**) Front and back views of a single fruiting body. (**B**,**D**) Front and back views of a clustered fruiting body.

During facility cultivation, developmental disorders such as blocked primordium differentiation and incomplete fruiting occurred frequently, severely restricting its large-scale production. Therefore, based on the initial screening of the GL2 strain in facility cultivation, a factory-scale mushroom cultivation experiment was further conducted to verify the agronomic traits and production adaptability of this strain under controllable conditions. The results are shown in Table 6 and Figure 7.

GL2 exhibited excellent agronomic traits under factory cultivation conditions. The fresh weight of the single bag was 80.46 ± 10.97 g, and the fresh weight of a single piece was 33.99 ± 8.20 g; both reached a relatively high level, indicating that this strain had good consistency and yield stability in the factory environment. Secondly, the cap length and width were 78.26 ± 12.07 mm and 110.87 ± 14.65 mm, respectively, with a cap width-to-length ratio of approximately 1.42. This indicated that the fruiting body morphology was relatively wide and elliptical, meeting the requirements of commercial *Ganoderma lucidum* for wide and symmetrical caps. The flesh thickness was 12.06 ± 2.71 mm, and the flesh was relatively thick, which is conducive to improving processing quality and product grade. In terms of the fruiting stalk characteristics, the diameter and length were 15.72 ± 4.90 mm and 52.39 ± 14.40 mm, respectively, conforming to the typical “long and thick stalk” morphological characteristics. Overall, GL2 exhibited a combination of characteristics including a wide cap, thick flesh, and a long and thick stalk in factory cultivation. These morphological and yield-related traits indicate that GL2 had good adaptability to factory-scale cultivation and considerable potential as a candidate strain for the industrial production of *G. leucocontextum*.

### 3.7. Nutritional Component Evaluation

#### 3.7.1. AAC

A comparative analysis of the AAC of the three types of *Ganoderma lucidum* was conducted, and the results are shown in Table 7. Except for methionine, the AAC in GL2 was significantly higher than that in ‘Fuzhi 94’ and ‘Wuzhi 8’ (*p* < 0.05). Specifically, the total amino acid (TAA) content in GL2 was 9.29 g/100 g, which was 2.27-fold and 1.64-fold higher than those of ‘Fuzhi 94’ and ‘Wuzhi 8’, respectively, and the essential amino acid (EAA) content in GL2 was 3.20 g/100 g, approximately 1.86-fold and 1.42-fold higher than those of the two controls. Regarding taste amino acids, GL2 showed higher contents of umami amino acids (4.23 g/100 g, 45.53 g/100 g of TAA) and sweet amino acids (2.15 g/100 g, 23.14% of TAA) compared to the two controls. Conversely, the proportion of bitter amino acids in GL2 (30.57 g/100 g) was lower than that in ‘Fuzhi 94’ (33.35 g/100 g) and ‘Wuzhi 8’ (31.92 g/100 g). Notably, the glutamic acid and aspartic acid contents in GL2 were particularly prominent (1.47 g/100 g and 1.01 g/100 g, respectively), contributing to its excellent flavor characteristics. In terms of pharmacological amino acids, the total content of glutamic acid, arginine, and aspartic acid in GL2 was 3.17 g/100 g (34.12 g/100 g of TAA), which was significantly higher than that of ‘Fuzhi 94’ (25.92 g/100 g) and ‘Wuzhi 8’ (28.04 g/100 g), suggesting that GL2 has higher health potential in liver protection, cardiovascular health, and immune regulation [37]. 

#### 3.7.2. Active Components

Polysaccharides are one of the most important active components of *Ganoderma lucidum*. As shown in Table 8, among the three samples, ‘Fuzhi 94’ had the highest PC (3.41 g/100 g), followed by GL2 (1.89 g/100 g) and ‘Wuzhi 8’ (1.14 g/100 g). Triterpenes and sterols are important sources of bitterness and various pharmacological activities in *Ganoderma lucidum*. The TTC and SC in GL2 were the highest (1.04 g/100 g), which was 1.73 times that of ‘Fuzhi 94’ (0.60 g/100 g) and 1.53 times that of ‘Wuzhi 8’ (0.68 g/100 g). Studies have shown that the TTC and SC of *G. leucocontextum* are approximately 20% higher than those of *Ganoderma lucidum*, which is consistent with the results of this study [38]. TPC and TFC are important antioxidant active components in Ganoderma lingzhi. The TPC of GL2 (0.37 g/100 g) was much higher than that of ‘Wuzhi 8’ (0.30 g/100 g) and ‘Fuzhi 94’ (0.26 g/100 g). The TFC of GL2 (0.12 g/100 g) was also significantly higher than those of the other two strains (*p* < 0.05). Existing studies have shown that the TPC and TFC of different *G. lingzhi* varieties vary greatly. The outstanding performance of GL2 in TFC in this study indicated that *G. leucocontextum* has a significant variety advantage in the accumulation of flavonoids. ‘Fuzhi 94’ had the highest PC, while GL2 performed optimally in TTC and SC, TPC, and TFC. In summary, the advantages of GL2 in multiple key active indicators further indicated that *G. leucocontextum* has the potential to be a high-value medicinal fungus. 

## 4. Discussion

Phylogenetic analysis based on ITS sequences showed that the seven *Ganoderma leucocontextum* strains were distributed across multiple subclades. The confrontation culture test further revealed three distinct types of antagonistic reactions, while ISSR analysis separated the seven strains into four genetic groups, with similarity coefficients ranging from 0.31 to 0.81. Notably, GL2 formed an independent branch and showed the lowest similarity coefficient (0.31) with the other strains, suggesting a relatively distinct genetic background. Mei et al. [39] showed that the ISSR analysis of 10 *Ganoderma lucidum* strains had a similarity coefficient range of 0.49–0.81. The similarity coefficient in this study is lower, which is speculated to be related to the wide geographical range of the strain sources. Lotfi et al. [40] also found that geographical isolation is an important driving factor for intraspecific genetic differentiation in the *Ganoderma* species. The multi-dimensional evidence collectively confirms that the tested *G. leucocontextum* strains have significant intraspecific genetic diversity.

Luangharn et al. [41] first reported the domestication and cultivation of wild *G. leucocontextum* in Yunnan, China. The biological efficiency of non-land-covered cultivation was 13.60% (58.18 g/kg). In this study, the fresh weight of GL2 in the factory cultivation mode was 80.46 g (corresponding to a biological efficiency of 21.28%), which was significantly better than its non-land-covered cultivation mode, indicating that through optimizing the substrate formula and environmental parameters, the yield can be effectively increased under southern facility cultivation conditions. Sun et al. [42] conducted a cultivation comparison of six *Ganoderma lucidum* strains in Lhasa, Tibet, China. The results showed that the individual mass of the *G. leucocontextum* strain 26 was 82.01 g, the cap diameter was 16.00 cm, and the biological efficiency was 23.60%. In terms of the uniformity of fruiting bodies, the above studies pointed out that *G. leucocontextum* has uniform fruiting bodies, high luster, and excellent agronomic traits. This is consistent with the typical characteristics of GL2 in factory cultivation, such as a wide cap, thick flesh, and a thick stalk. On the other hand, Wu et al. [43] selected L4968 ‘Yunling 3’ from the *G. leucocontextum* strain L4439 as a control and successfully bred it. Under the cultivation conditions in Yunnan, China, the two strains had complete fruiting body morphology, normal cap splitting, and excellent agronomic traits. Among them, the fresh weight of L4968 in a single bag was 83.94 g, with a biological efficiency of 15.24%, while in this study, GL3 (L4439) could form fruiting bodies, but the cap splitting was hindered, and the commercial traits were poor; GL6 (L4968) only stagnated at the primordium stage and failed to further develop. The other strains GL4, GL5, and GL7 in the study all had fruiting body obstacles under factory cultivation. This indicates that the normal development of *G. leucocontextum* fruiting bodies is highly sensitive to ecological conditions (temperature, humidity, light, altitude, etc.).

To further evaluate the nutritional and functional value of GL2, in this study we compared the AAC and active component content of its factory-grown fruiting bodies with those of *Ganoderma lucidum* (‘Fuzhi 94’) and *Ganoderma sinense* (‘Wuzhi 8’). From the perspective of AAC, the TAA content of GL2 was the highest among the three *Ganoderma* species, followed by *G. sinense*, and the lowest was *G. lucidum*. This shows that GL2 has a relative advantage in protein nutritional value. However, the content of methionine (Met) in GL2 was only 0.02 g/100 g, significantly lower than that of *G. sinense* (0.10 g/100 g) and *G. lucidum* (0.11 g/100 g). The proportion of bitter amino acids in GL2 was relatively low, while the proportions of sweet and savory amino acids were relatively high, indicating that GL2 may present a more harmonious, fresh, and refreshing taste characteristic, with a relatively weakened bitter perception. This enables it to achieve better sensory acceptance when developing functional foods (such as reishi beverage, soup packs, meal replacement powder, etc.). In addition, the content of GL2’s therapeutic amino acids (glutamic acid, arginine, aspartic acid) is 3.17 g/100 g, accounting for 34.12 g/100 g of the TAA, which is significantly higher than that of *G. lucidum* and *G. sinense*. Based on the above active ingredients and amino acid indicators, GL2 demonstrates nutritional and functional advantages in multiple aspects. Its outstanding high total phenols and high triterpenoids characteristics give this strain unique comprehensive utilization potential.

In terms of active ingredients, the PC of GL2 is 1.89 g/100 g, significantly higher than that of *G. sinense* (1.14 g/100 g), but lower than that of *G. lucidum* (3.41 g/100 g). The significant differences in PC among different species of *Ganoderma* have been widely reported. This difference is mainly attributed to the combined influence of species’ genetic background and cultivation conditions [44]. Wang et al. [16] found that the TCC of *G. leucocontextum* is about 20% higher than that of *G. lucidum*, with stronger polysaccharide activity, and the higher protein content may be one of the reasons for its less bitter taste. In this study, the TTC and SC of GL2 (1.04 g/100 g) are significantly higher than those of *G. lucidum* (0.60 g/100 g) and *G. sinense* (0.68 g/100 g), which is consistent with the above species superiority conclusion. However, the PC (1.89 g/100 g) of GL2 is lower than that of *G. lucidum* (3.41 g/100 g), which is different from the result reported by Wang et al. [16] that the PC of *G. leucocontextum* is higher than that of *G. lucidum*. This may be related to the strain source and cultivation substrate. Particularly notable is that the TTC and SC of GL2 are as high as 1.04 g/100 g, ranking first among the three species in this study. This is consistent with the result found by Peng et al. [12] in the systematic comparison of *G. leucocontextum* and *G. lucidum*, suggesting that *G. leucocontextum* has obvious genetic superiority in terms of triterpenoid-based therapeutic active substances. In terms of TPC and TFC, the TPC of GL2 (0.37 g/100 g) is significantly higher than that of *G. lucidum* (0.26 g/100 g) and *G. sinense* (0.30 g/100 g). Heleno et al. [45] found that phenolic compounds are the main contributors to the antioxidant activity of *Ganoderma* species. Therefore, GL2, being rich in phenolic compounds, may possess potential as a functional food ingredient. However, specific antioxidant activity assays and interaction studies among components are required to confirm this in future research.

In terms of mycelial antagonism ability, the inhibition rates of the seven strains range from 51.28% to 68.79%, with an overall level that is relatively close. It is speculated that they all belong to the medium resistance level. This result indicates that the tested *G. leucocontextum* strains have not yet screened out strong-resistant strains in terms of resisting mycelial infection, but there are still certain amplitude differences among the strains. Therefore, in practical production, it is recommended to combine with substrate optimization, environmental regulation, and other comprehensive management measures to effectively control the risk of mycelial contamination.

## 5. Conclusions

In conclusion, GL2 shows high yield potential and excellent commercial traits under facility and factory cultivation conditions, and has good application prospects. Based on the results of multiple dimensions such as genetic background, physiological characteristics, mushrooming performance, active ingredients, and resistance evaluation, the GL2 strain, with its unique genetic background, excellent yield performance, good commercial appearance, and outstanding composition of active ingredients, exhibits high-yield and high-quality traits under the facility and factory cultivation conditions in Fujian, China. It can be promoted as an excellent cultivation strain for *Ganoderma leucocontextum* in the southern region and verified as feasible for replacing traditional *Ganoderma lingzhi* (*Ganoderma lucidum* and *Ganoderma sinense*) as functional medicinal and food raw materials. However, there are still certain limitations: on the one hand, no multi-batch pilot production has been carried out in the factory, and it is impossible to systematically evaluate the yield stability and quality consistency of this strain under different seasons and substrate batch conditions; on the other hand, the genetic breeding research on enriching important active components such as triterpenoids is still a blank. Subsequent research will focus on the establishment of the factory cultivation technology system of GL2 to provide technical scheme references for the large-scale promotion of *G. leucocontextum* in the southern region. At the same time, by leveraging the unique genetic background of GL2 as an independent branch, we aim to identify specific functional gene clusters related to high triterpenoid content and conduct molecular marker-assisted breeding. The goal is to select new strains of *G. leucocontextum* that are adapted to the southern cultivation environment and possess both high-yield and high-quality characteristics.

## Figures and Tables

**Table 1:** Sources of 7 *Ganoderma leucocontextum* Strains.

Strains	Original Strain Code	Source
GL1	*Ganoderma leucocontextum* 1	Nyingchi, Tibet Autonomous Region, China
GL2	*Ganoderma leucocontextum* 2	Nyingchi, Tibet Autonomous Region, China
GL3	4439	Biotechnology and Germplasm Resources Institute, Yunnan Academy of Agricultural Sciences, Yunnan, China
GL4	4914	Biotechnology and Germplasm Resources Institute, Yunnan Academy of Agricultural Sciences, Yunnan, China
GL5	4966	Biotechnology and Germplasm Resources Institute, Yunnan Academy of Agricultural Sciences, Yunnan, China
GL6	4968	Biotechnology and Germplasm Resources Institute, Yunnan Academy of Agricultural Sciences, Yunnan, China
GL7	*Ganoderma leucocontextum*	Yunnan Yunjun Technology (Group) Co., Ltd., Kunming, Yunnan, China

**Table 2:** ISSR primer sequences.

Primers	Primer Sequence (5′→3′)	Primers	Primer Sequence (5′→3′)
ISSR1	CACCACACACACACACA	ISSR11	AAGAAGAAGAAGAAGAAGC
ISSR2	AGAGAGAGAGAGAGAGT	ISSR12	CACGAGAGAGAGAGAGA
ISSR3	GAGAGAGAGAGAGAGAC	ISSR13	TTCCCTTCCCTTCCC
ISSR4	CACACACACACACACAT	ISSR14	GAGAGAGAGAGAGAGAT
ISSR5	ACACACACACACACACT	ISSR15	AGTGTGTGTGTGTGT
ISSR6	GAGAGAGAGAGAGAGACT	ISSR16	GGATGCAACACACACACAC
ISSR7	GAGAGAGAGAGAGAGACC	ISSR17	TGCACACACACACAC
ISSR8	ACAACACACACACACAC	ISSR18	GTGACACACACACAC
ISSR9	AGAGAGAGAGAGAGAGC	ISSR19	CGTGTGTGTGTGTGT
ISSR10	AGCAGCAGCAGCAGCAGCG	ISSR20	AGTGTGTGTGTGTGT

**Table 3:** Antagonistic reaction among the seven *G. leucocontextum* strains.

Strains	GL1	GL2	GL3	GL4	GL5	GL6	GL7
GL1	-						
GL2	+++	-					
GL3	+	++	-				
GL4	+	+	++	-			
GL5	++	+++	++	++	-		
GL6	++	+	+++	+	+	-	
GL7	++	++	+++	++	++	++	-

Notes: “+++” indicates a protrusion-type reaction, “++” indicates a furrow-type reaction, “+” indicates a linear-type reaction, and “-” indicates no antagonistic reaction.

**Table 4:** Inhibition rates of *Trichoderma* on seven *G. leucocontextum* strains.

Strains	Inhibition Rate (%)
GL1	-
GL2	63.49 ± 2.94 a
GL3	51.28 ± 2.49 b
GL4	68.42 ± 3.99 a
GL5	66.28 ± 2.66 a
GL6	68.79 ± 1.09 a
GL7	59.84 ± 5.49 ab

Notes: ”-” Indicates not detected. Means with different letters indicate significant differences (*p* < 0.05).

**Table 5:** Time to full bag colonization and primordium formation of seven *G. leucocontextum* strains.

Strains	Days to Full Bag Colonization (d)	Days to Primordium Formation (d)
GL1	35	38
GL2	30	39
GL3	31	38
GL4	35	45
GL5	35	45
GL6	33	45
GL7	35	40

**Table 6:** Agronomic traits of GL2 under industrial cultivation.

Cap Length (mm)	Cap Width (mm)	Flesh Thickness (mm)	Stipe Diameter (mm)	Stipe Length (mm)	Fresh Weight per Bag (g)	Fresh Weight per Individual Fruiting Body (g)
78.26 ± 12.07	110.87 ± 14.65	12.06 ± 2.7	15.72 ± 4.9	52.39 ± 14.40	80.46 ± 10.97	33.99 ± 8.20

**Table 7:** Amino acid content of different fruiting bodies.

AAC (g/100 g)	GL2	Fuzhi 94	Wuzhi 8
His	0.40 ± 0.001290 a	0.24 ± 0.000296 c	0.25 ± 0.001370 b
Tyr	0.23 ± 0.001827 a	0.08 ± 0.000120 c	0.12 ± 0.001111 b
Ile *	0.37 ± 0.002060 a	0.17 ± 0.001774 c	0.24 ± 0.002640 b
Val *	0.49 ± 0.001533 a	0.23 ± 0.000556 c	0.32 ± 0.000058 b
Ala	0.62 ± 0.001543 a	0.28 ± 0.001290 c	0.41 ± 0.000439 b
Pro	0.54 ± 0.001999 a	0.21 ± 0.002079 c	0.36 ± 0.002322 b
Arg	0.69 ± 0.001217 a	0.15 ± 0.001863 c	0.26 ± 0.001058 b
Lys *	0.57 ± 0.000450 a	0.20 ± 0.002086 c	0.33 ± 0.002235 b
Ser	0.56 ± 0.001583 a	0.27 ± 0.001843 c	0.36 ± 0.002434 b
Glu	1.47 ± 0.001572 a	0.47 ± 0.000415 c	0.72 ± 0.001505 b
Gly	0.49 ± 0.002250 a	0.24 ± 0.001590 c	0.33 ± 0.005186 b
Met *	0.02 ± 0.000096 c	0.11 ± 0.000262 a	0.10 ± 0.000222 b
Leu *	0.86 ± 0.001963 a	0.52 ± 0.000640 c	0.62 ± 0.000684 b
Phe *	0.41 ± 0.000640 a	0.18 ± 0.002118 c	0.27 ± 0.001082 b
Thr *	0.56 ± 0.000716 a	0.30 ± 0.000077 c	0.37 ± 0.002224 b
Asp	1.01 ± 0.001386 a	0.44 ± 0.000896 c	0.61 ± 0.001663 b
Phe + Tyr	0.64 ± 0.001528 a	0.27 ± 0.002000 c	0.39 ± 0.000577 b
EAA	3.2 ± 0.0023099 a	1.72 ± 0.005000 c	2.25 ± 0.003215 b
NEAA	4.69 ± 0.004359 a	1.91 ± 0.000577 c	2.79 ± 0.007234 b
TAA	9.29 ± 0.002000 a	4.09 ± 0.001528 c	5.67 ± 0.002517 b

Note: The different lowercase letters of the same detection component indicate significant differences (*p* < 0.05); *: Essential amino acids; EAA: Essential amino acids; NEAA: Non-essential amino acids; TAA: Total amino acids.

**Table 8:** Scale of active components in fruiting bodies.

Strains	PC (g/100 g)	TTC and SC (g/100 g)	TPC (g/100 g)	TFC (g/100 g)
GL2	1.89 ± 0.130512 b	1.04 ± 0.064501 a	0.37 ± 0.000534 a	0.12 ± 0.000265 a
Fuzhi 94	3.41 ± 0.320052 a	0.60 ± 0.056000 b	0.26 ± 0.002221 c	0.11 ± 0.001000 b
Wuzhi 8	1.14 ± 0.101002 c	0.68 ± 0.057000 b	0.30 ± 0.010616 b	0.10 ± 0.001587 c

Means with different letters indicate significant differences (*p* < 0.05).

## Data Availability

The original contributions presented in the study are included in the article; further inquiries can be directed to the corresponding author.
